# Exploring predictors of analgesic response in adult women regularly using NSAIDs: insights from a machine learning approach

**DOI:** 10.3389/fpain.2026.1878751

**Published:** 2026-07-29

**Authors:** Christopher Huong, Laura C. Seidman, Kevin M. Hellman, Laura A. Payne

**Affiliations:** 1 McLean Hospital, Belmont, MA, United States; 2 Endeavor Health, Chicago, IL, United States; 3 University of Chicago, Chicago, IL, United States; 4 Harvard Medical School, Boston, MA, United States

**Keywords:** drug response, dysmenorrhea, menstrual pain, menstrual symptoms, non-steroidal anti-inflammatory drugs, machine learning

## Abstract

**Introduction:**

Non-steroidal anti-inflammatory drugs (NSAIDs) are considered a first-line treatment for dysmenorrhea (menstrual pain). However, as much as 20% of women fail to achieve sufficient pain relief from NSAIDs, although factors associated with NSAID non-response are not well-known. The purpose of this study was to investigate factors associated with analgesic response to NSAIDs for dysmenorrhea.

**Methods:**

Data were from a large survey study of adult women who reported regularly using NSAIDs for dysmenorrhea. Demographic information, menstrual characteristics, comorbid conditions, and psychosocial factors were collected. NSAID non-response was defined as self-reported minimal or no menstrual pain relief from NSAIDs. We repeatedly applied a group least absolute shrinkage and selection operator (LASSO) logistic regression 100 times, and averaged results to account for model uncertainty and to identify variables related to NSAID non-response.

**Results:**

Of 512 women (mean age = 34.0 years, SD = 9.4) who met inclusion criteria, 182 (35.5%) were classified as NSAID non-responders. NSAID non-responders reported significantly higher average menstrual pain severity than responders (mean = 7.4 vs. 6.2 on a 0–10 scale, *p* < 0.001). The strongest factors associated with NSAID non-response included greater menstrual pain severity and more severe menstrual-related symptoms (e.g., bloating and dull pelvic pain), as well as greater sleep disturbance. Higher negative affect was modestly associated with better odds of NSAID response. Classical risk factors such as heavy menstrual bleeding and self-reported endometriosis were not significantly linked to NSAID response in this sample. However, the LASSO models achieved only modest discrimination (mean cross-validated AUC = 0.64), indicating considerable unexplained variability.

**Discussion:**

In this large cross-sectional cohort, women reporting inadequate menstrual pain relief from NSAIDs had more severe menstrual pain and related symptoms, along with indicators of pain amplification (sleep disturbance). Most psychosocial factors (e.g., negative mood) did not correlate with NSAID non-response. These findings underscore the heterogeneity of dysmenorrhea and suggest that clinical factors alone may be insufficient to predict NSAID effectiveness. Further prospective studies are needed to confirm these associations and to explore whether interventions (such as optimizing NSAID timing or addressing sleep disturbances) could improve menstrual pain outcomes for those with NSAID non-response.

## 1 Introduction

Dysmenorrhea, or menstrual pain, is extremely common, affecting 16%–91% of reproductive-aged girls and women (1), and severely affecting approximately 10%–20% of girls and women to the extent of regularly missing school or work (2, 3). Although dysmenorrhea sometimes (∼20%) appears to be caused by anatomical issues like endometriosis and fibroids, most often (∼80%) there are no clearly identifiable problems other than uterine contractions (4). Although it is believed that excess levels of prostaglandins cause increased uterine contractions, inflammation, and pain, other mechanisms, including alterations in the central nervous system, also seem to be involved, suggesting multiple pathways that may be responsible for the experience of menstrual pain (5–8).

Notably, the inhibition of prostaglandin synthesis by nonsteroidal anti-inflammatory drugs (NSAIDs) is considered the first-line treatment for dysmenorrhea. A review of 35 randomized controlled trials estimated an odds ratio of 4.37 for pain relief when comparing NSAIDs to placebo (9), suggesting that those who take NSAIDs were approximately four times more likely to experience moderate or excellent pain relief. However, pain relief is most often incomplete (10, 11). A review of 51 papers estimated that 18% of women with dysmenorrhea experience minimal or no relief from NSAIDs (12).

Reasons for non-responsiveness to NSAIDs for menstrual pain are unclear and may differ based on underlying pathophysiology (6). One study in dysmenorrhea found that women with minimal pain relief had significantly lower serum naproxen levels, implicating suboptimal NSAID bioavailability as one potential mechanism (13). Also, anatomical problems (e.g., endometriosis) could render prostaglandin-focused therapies less effective. Studies suggest that NSAIDs are less effective for menstrual pain in those with endometriosis or fibroids (14). It is also plausible that central nervous system sensitization contributes to NSAID non-response (15). Sensory testing in endometriosis and some dysmenorrhea patients reveals signs of altered central sensitization compared to pain-free participants (10, 16). As NSAIDs primarily target peripheral rather than central pain processes (17), we may expect markers of central sensitization (e.g., pain sensitivity, comorbid chronic pain conditions) to be associated with poorer response to NSAIDs to the extent that central processes are important drivers of menstrual pain.

Given the biopsychosocial nature of dysmenorrhea (18), a wide range of factors are likely involved in the experience of menstrual pain, including psychosocial concerns such as perceived stress and social support (1, 19). It is thus plausible that psychosocial factors may also moderate the effects of analgesic drugs, contributing to the variability in NSAID efficacy, though little is yet known concerning this hypothesis (20). Identifying patient-level variables associated with NSAID non-response could point to underlying mechanisms and risk factors for refractory dysmenorrhea and help clinicians recognize those who may need alternative or adjunct treatments. For example, the emergence of digital tracking tools for reproductive health have shown promise in providing accessible and individualized management of menstrual pain and related symptoms (21). These digital tools can be complemented by statistical methods that inform the selection of questions (variables) to include in the assessments used to predict meaningful clinical outcomes (e.g., analgesic response to specific symptoms).

In the present study, we took an exploratory, data-driven approach to examine associations with NSAID non-response in regularly-menstruating adults. We analyzed data from a large online survey of women who reported using NSAIDs for menstrual pain, employing a machine-learning approach to variable selection (i.e., group LASSO regularization) to handle a broad array of potential predictors. Given the exploratory nature of this study and dearth of knowledge in this area, no specific *a priori* hypotheses were proposed. Our goals were to identify demographic, clinical, or psychosocial variables related to self-reported NSAID non-response, and to suggest the most consistent hypotheses for why some women do not experience adequate pain relief from NSAIDs.

## 2 Methods

### 2.1 Participants

This analysis utilized data (*n* = 1,232) from a large cross-sectional survey of women's health, described in detail elsewhere (22, 23). In brief, participants were recruited from a national research panel (Market Cube Inc., now Sago Health, Iselin, NJ) via online invitations, with sampling stratified to approximate the U.S. population age distribution. Eligible respondents were females ages 18–55 who had experienced at least one menstrual period in the past 3 months and had regular menstrual cycles in the past year. Women who were currently pregnant or who could not read English were excluded. To ensure a range of dysmenorrhea severity, enrollment was stratified by self-reported typical menstrual pain on a 0–10 numeric rating scale (NRS), aiming for approximately 25% of respondents with mild or no pain (0–2), 60% with moderate pain (3–7), and 15% with severe pain (8–10) (24). For the present study, only the subset of respondents who indicated they usually take “NSAIDs (such as ibuprofen, naproxen, mefenamic acid, etc.)” for their menstrual pain were analyzed.

### 2.2 Procedures

**Figure 1:**
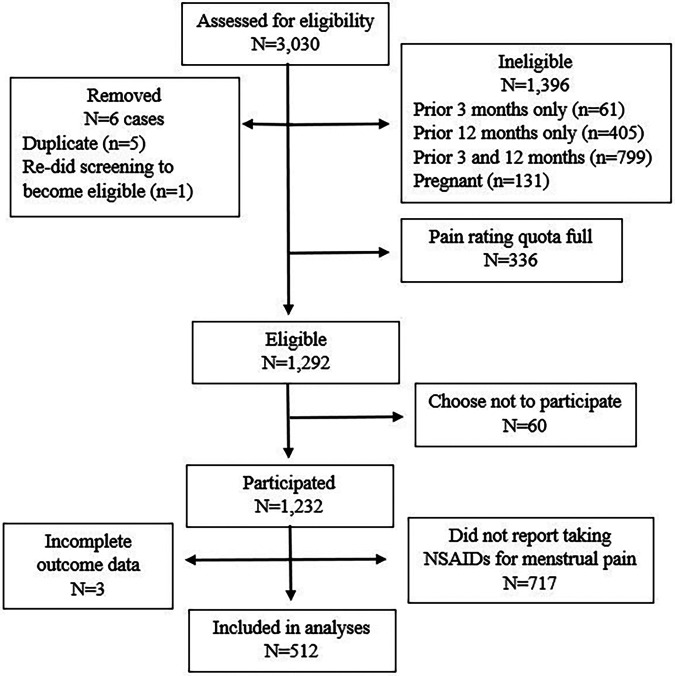
Enrollment flow diagram.

Interested potential participants who clicked the link were directed to a screening page assessing eligibility criteria. Eligible potential participants were directed to a study information sheet; continuing with the survey was considered agreement to participate. Ineligible participants were redirected to a Market Cube webpage. The survey was housed on REDCap (Research Electronic Data Capture (25, 26), a secure survey administration platform on the institution's servers. Participants who completed the survey were compensated 200 points (equivalent to $2) to their accounts with the panel company. See Figure 1 for a recruitment and enrollment flowchart.

#### 2.2.1 Ethical approval and informed consent

The study procedures were conducted in accordance with the Declaration of Helsinki and was determined to meet criteria for exemption by the Mass General Brigham IRB under protocol #2020P002578. All participants provided informed consent electronically to participate in the study.

### 2.3 Primary outcome

The outcome of interest was medication efficacy, presented as “How well does the medication usually work?” (1 = “Not at all”, 2 = “Somewhat”, 3 = “Moderately well”, 4 = “Very well”, 5 = “Works completely”, which in context of the specified subset, we considered to represent the efficacy of NSAIDs for alleviating menstrual pain. For simplicity and model interpretability, we recoded the outcome to a binary variable, with responses of “1” (Not at all) and “2” (Somewhat) indicating non-response, and “3” through “5” indicating response to NSAIDs.

### 2.4 Predictive measures

The complete set of predictors included in the data analysis is detailed in the Supplementary Materials. Briefly, these included demographic variables (e.g., age, race, education), menstrual variables (e.g., age of menarche, average menstrual pain severity, presence and severity of specific menstrual symptoms (27), chronic pain variables, chronic health conditions, medications, and psychological variables.

Several psychosocial constructs were assessed in the survey. The Menstrual Sensitivity Index (MSI (23), was used to assess fear and anxiety related to physical symptoms caused by menstruation, where higher scores indicate higher sensitivity (full sample standardized *α* = 0.91). The Pain Catastrophizing Scale (PCS (28) total score and subscales were used to assess pain-related general catastrophizing, rumination, magnification, and helplessness (total score *α* = 0.97). The Positive and Negative Affect Scale (PANAS (29) was used to assess positive and negative affect over the past week (*α* = 0.92 and 0.94 for positive and negative affect, respectively). The 10-item Perceived Stress Scale (PSS-10 (30) was used to assess feelings of participant's lives as unpredictable and overwhelming (*α* = 0.70). The Anxiety Sensitivity Index-3 (ASI-3 (31) is an 18-item measure used to assess global anxiety sensitivity (i.e., fear of anxiety-related sensations due to beliefs of harmful consequences) and it's three dimensions: physical, cognitive, and social concerns (*α* = 0.96, 0.92, 0.94, and 0.88, respectively). Lastly, Patient Reported Outcomes Measurement Information System (PROMIS (32) short forms were used to assess anxiety (*α* = 0.96 (33), depression (*α* = 0.97 (33, 34), pain interference (*α* = 0.97 (35), physical function (*α* = 0.95 (36), emotional support (*α* = 0.97 (37), companionship (*α* = 0.95 (37), fatigue (*α* = 0.96 (38), and sleep disturbance (*α* = 0.87 (39, 40).

When multi-categorical variables had highly unbalanced distributions (i.e., one or more categories with <1% response frequency), we collapsed them into fewer categories. If ordinal scales had five or more responses, we coded them as continuous variables. Further, we standardized all continuous variables prior to final modeling (i.e., mean = 0, standard deviation = 1). We compared the NSAID responder and non-responder groups on these characteristics using appropriate univariate tests (chi-square tests for categorical variables and Welch's *t*-tests for continuous variables).

### 2.5 Data analysis

We use a least absolute shrinkage and selection operator (LASSO) regularization approach to perform variable selection among many simultaneous predictors while attenuating issues of multicollinearity and overfitting (41). LASSO regularization adds an L1 penalty term to the regression equation to shrink estimated coefficients towards zero (42), thereby improving generalizability by reducing the degree that the model overfits to the idiosyncratic noise of the data. The LASSO has the advantage over some other regularization methods in that it can shrink coefficients to exactly zero if they do not improve the predictive performance of the model, and thus automatically performs variable selection (predictors with non-zero coefficient estimates are “selected” into the model) and produces a parsimonious set of important variables (43). As several of our predictors were categorical variables with multiple levels (e.g., education levels coded as “Graduate Degree”, “Bachelor's Degree”, and “No Degree”, which are typically dummy coded into k-1 variables, we opted for the group LASSO extension, as described by Yang & Zuo (44) and implemented in R package *gglasso* (45), to allow groups of dummy variables to be included or excluded together, facilitating interpretability (46). As we recoded the outcome to a binary variable, we used a logistic regression model (with group LASSO regularization) to estimate the conditional probability of NSAID efficacy given a set of predictors.

The L1 penalty term is controlled by the tuning parameter *λ*, which influences the degree of shrinkage of the coefficient estimates. When *λ* = 0, the LASSO reduces to a standard regression model and no predictors are excluded, and when *λ* is very large, all coefficients are shrunk to zero resulting in a null model. To select optimal *λ* values, we use 10-fold cross-validation, where the data is randomly partitioned into 10 equal-sized “folds”. Multiple group LASSO models with a range of different *λ* values are fit to the data from nine folds, and their predictions are tested against the remaining fold. This is repeated for all folds, and the *λ* value that has the lowest average classification error is retained for the final model.

Given that *λ* may vary depending on the random partitions of the cross-validation procedure, we opted to use a similar approach to Han and colleagues (47) by repeating the group LASSO with cross-validation 100 times, which results in slightly different *λ* values, and thus potentially different coefficient estimates for each model. For each variable we calculate the proportion of models in which it was included (the “selection fraction”). We then averaged the non-zero coefficients across the 100 models to obtain the mean coefficient (log-odds) for each variable, which were exponentiated to produce an odds ratio (OR). To assess how well the cross-validated models discriminate between NSAID responders and non-responders, we computed the area under the receiver operating characteristic curve (AUC). The AUC ranges from 0 to 1, with 0.50 being no better than chance, and 1.0 being perfect model predictions. Lambda values varied slightly across the 100 iterations due to differences in random fold assignment; averaging over iterations integrates over this variability.

#### 2.5.1 Missing data

Chi-square tests were used to assess if the probability of missingness in the predictor variables depended on the outcome. Missing data were then imputed using the random forest method as described by Stekhoven and Bühlmann (48), to handle mixed data (i.e., continuous and categorical variables). All item-level variables of the full sample were included in the imputation step, then used to compute mean scores for each psychometric scale, which were included in further analysis. Imputation accuracy was assessed using out-of-bag error estimates, where model-predicted values are compared to true values of the data recursively held out during model fitting: normalized root mean square error (NRMSE) for continuous variables, and proportion of falsely classified entries (PFC) for categorical variables. For the LASSO models, we retained those who endorsed using NSAIDs for pain during their period and who had no missingness for the outcome variable, leading to an analytic sample of *n* = 512.

#### 2.5.2 Sensitivity analysis

To explore if changing the cut off used to recode the outcome would alter results, we repeat the analysis with recoding responses “1” through “3” to indicate poor NSAID response, and responses “4” and “5” to indicate high NSAID response.

To explore whether a non-linear variable selection method would yield similar results to the LASSO models, we use the Boruta algorithm, implemented with the R package *Boruta* (49), The Boruta algorithm is a wrapper built around random forests to provide estimates of predictor importance, and returns a decision on whether each variable significantly improves predictive accuracy above random permutations of the predictors. We compare the set of variables selected by the Boruta algorithm with the set of variables selected by the group LASSO models.

## 3 Results

### 3.1 Sample characteristics

Of the 1,232 participants in the survey, 512 met inclusion criteria and had complete outcome data for analyses (Table 1). Full descriptive statistics for both non-imputed and imputed data sets can be found in the Supplementary Materials. The final predictor set was 116 variables. When accounting for each level of multi-category variables, 196 parameters were to be estimated by the model, excluding the intercept. Missingness was not found to be significantly related to NSAID across all predictor variables, and thus missing data were considered to be missing at random. The random forest imputation for missing data yielded adequate error estimates when comparing imputed values to true out-of-bag values, with imputed continuous variables deviating by 30% on average, and 9% of imputed categorical variables misclassified (NRMSE = 0.30, PFC = 0.09). Variables with higher rates of missing data were concentrated among body-region pain indicators and specific medication items; the majority of these were not selected by the final LASSO models or the Boruta analysis. Among the seven variables with selection fractions of 1.00 in the primary LASSO analysis, missing rates were low, limiting the influence of imputation uncertainty on the primary findings.

**Table 1:** Descriptive statistics of the analytic sample before missing data imputation.

Variable [M (SD) unless otherwise noted]	Total (*N* = 512)	NSAID Responder (*N* = 330)	NSAID Non-Responder (*N* = 182)	*p*-value*
Age (years)	34.0 (9.4)	33.8 (9.5)	34.3 (9.3)	0.578
PCS total, mean item score (0–4)	1.6 (1.1)	1.5 (1.1)	1.63 (1.1)	0.286
MSI mean item score (0–5)	2.8 (1.2)	2.6 (1.3)	3.1 (1.1)	<0.001
Menstrual pain severity (0–10)	6.6 (2.0)	6.2 (2.0)	7.4 (1.6)	<0.001
Menstrual symptom severity in past 6 months (0–10)				
Abdominal cramps	6.8 (2.3)	6.4 (2.4)	7.6 (1.7)	<0.001
Dull abdominal pain or discomfort	6.4 (2.3)	5.9 (2.5)	7.3 (1.6)	<0.001
Low back pain	5.8 (3.0)	5.4 (3.0)	6.7 (2.7)	<0.001
Headache or migraine	5.7 (3.0)	5.3 (3.0)	6.5 (2.8)	<0.001
Aches all over	5.0 (3.1)	4.4 (3.1)	5.9 (2.8)	<0.001
Bloating	6.4 (2.7)	5.8 (2.8)	7.4 (2.1)	<0.001
Nausea	3.8 (3.3)	3.5 (3.2)	4.3 (3.2)	0.006
Diarrhea	3.8 (3.3)	3.6 (3.3)	4.1 (3.3)	0.091
More bowel movements	4.4 (3.2)	4.1 (3.1)	4.9 (3.1)	0.006
Pain in upper thighs	3.5 (3.4)	3.2 (3.3)	4.1 (3.5)	0.005
Vomiting	1.6 (2.7)	1.5 (2.7)	1.6 (2.7)	0.895
Reduced appetite	3.5 (3.3)	3.1 (3.2)	4.2 (3.4)	<0.001
Constipation	2.9 (3.2)	2.7 (3.1)	3.2 (3.2)	0.084
Fewer bowel movements	2.2 (3.0)	2.1 (2.9)	2.2 (3.0)	0.746
Education [n (%)]				
Less than Bachelor's degree	296 (57.8)	179 (54.2)	117 (64.3)	0.035
Bachelor's degree	144 (28.1)	98 (29.7)	46 (25.3)	0.336
Graduate degree	72 (14.1)	53 (16.1)	19 (10.4)	0.106
Race^a^ [n (%)]				
White	384 (75.0)	224 (73.9)	140 (76.9)	0.522
Black	65 (12.7)	44 (13.3)	21 (11.5)	0.656
Asian	31 (6.1)	22 (6.7)	9 (4.9)	0.556
American Indian or Alaskan Native	11 (2.1)	8 (2.4)	3 (1.6)	0.794
Native Hawaiian or other Pacific Islander	0 (0.0)	0 (0.0)	0 (0.0)	−
Multi-Racial	21 (4.1)	12 (3.6)	9 (4.9)	0.630

* *p*-values reflect unpaired two-sample Welch's *t*-tests and 2 × 2 chi-squared tests. Mean scores for PCS and MSI reflect the mean of individual item scores within each measure.

a In the analyses, race was collapsed into a binary variable of White/Non-White due to high imbalance.

Briefly, the sample was majority white (75.0%) with a mean age of 34.0 years old (SD = 9.4), and with less than half (42.2%) of the sample holding undergraduate or advanced degrees. Based on each participant's response to the NSAID efficacy question, 330 women (64.5%) were classified as NSAID responders (moderate to complete pain relief), whereas 182 women (35.5%) were classified as NSAID non-responders (no or minimal pain relief). The overall sample mean reported menstrual pain severity (on 0–10 NRS) was 6.6 (SD = 2.0), consistent with moderate-to-severe pain on average. Menstrual-related symptoms were reported on an 11-point scale (0 = “not present”, 10 = “extremely severe”, with the most severe symptoms including abdominal cramps (*M* = 6.8, SD = 2.3), abdominal pain (*M* = 6.4, SD = 2.3), bloating (M = 6.4, SD = 2.7), lower back pain (M = 5.8, SD = 3.0), and headaches (M = 5.7, SD = 3.0). Only 5.7% of the total sample reported having a physician diagnosis of endometriosis. Other chronic pain or health conditions were reported relatively infrequently, with the most common being migraine headaches (31.2%), back pain (29.9%), and digestive conditions (15.2%).

Univariate tests on non-imputed data reveal that NSAID responders were relatively similar to non-responders (Table 1). Women in the NSAID non-responder group reported more severe menstrual pain on average than responders (mean NRS 7.4 vs. 6.2, t(444.4) = 7.4, *p* < 0.001). Of the 14 possible menstrual symptoms, NSAID non-responders reported significantly higher severity (i.e., *p* < 0.01) on 10; abdominal cramps, dull abdominal pain or discomfort, low back pain, headache or migraine, aches all over, bloating, nausea, more bowel movements, pain in upper thighs, and reduced appetite. Demographic factors such as age, race, and income did not differ significantly between groups.

### 3.2 LASSO variable selection identifies seven variables reliably associated with NSAID response

Table 2 shows the variables selected by the group LASSO models with non-zero selection fractions (i.e., had a non-zero coefficient estimated by at least one of 100 group LASSO models) and the mean of non-zero coefficients, representing odds ratio of NSAID response. As continuous predictors were standardized prior to modeling, odds ratios can be interpreted as the multiplicative change in the odds of NSAID response corresponding to each standard deviation increase in the predictor. Thus, an estimated OR greater than 1 indicates that increases in that variable are associated with greater odds of being classified as an NSAID responder, while an OR of less than 1 indicates that a variable is associated with lower odds of being classified as an NSAID responder.

**Table 2:** Selection fraction and mean coefficients from 100 group LASSO models.

Variable name	Selection fraction	Mean coefficient (odds ratio)
Demographic variables		
Income	0.93	1.03
Number of adults living in your home	0.87	0.98
City: Large city	0.38	1.03
City: Small city	0.38	0.99
City: Suburbs of a large city	0.38	1.00
City: Rural area	0.38	0.98
Menstrual variables		
Period during the past 7 days	0.91	1.09
Number of periods in the past 12 months	0.96	1.11
Typical level of menstrual pain intensity	1.00	0.75
Menstrual symptom severity in past 6 months		
Dull abdominal pain or discomfort	1.00	0.82
Low back pain	1.00	0.95
Aches all over	1.00	0.94
Bloating	1.00	0.76
Vomiting	0.97	1.08
Fewer bowel movements	0.93	1.05
Reduced appetite	0.90	0.95
Headache or migraine	0.89	0.98
Diarrhea	0.87	1.02
Clinical variables		
Hormone product/supplement: None	0.89	0.96
Chronic pain condition: Back pain	0.91	0.94
PANAS negative affect subscale	1.00	1.30
PANAS positive affect subscale	0.97	1.03
PROMIS sleep disturbance	1.00	0.70
PROMIS pain interference	0.97	0.83
PROMIS physical function	0.93	1.06
ASI-3 physical concerns subscale	0.96	1.11
PCS magnification subscale	0.87	1.03

Bolded variables indicate selection fractions of 1.00.

Overall, 25 of 116 included variables had non-zero selection fractions and non-zero mean coefficients (having no chronic pain conditions had a selection fraction of 0.18, but a mean log-odds of 0.00 after rounding). Selection fractions ranged from 0.38 to 1.00, and ORs ranged from 0.70 to 1.30. AUC values were computed for each model with a mean value of 0.64 (standard deviatio*n* = 0.01), suggesting modest predictive performance. Variance inflation factor (VIF) values for the selected predictors ranged from 1.03 (number of adults living in your home) to 3.0 (PROMIS pain interference), suggesting minimal multicollinearity after LASSO variable selection.

**Figure 2:**
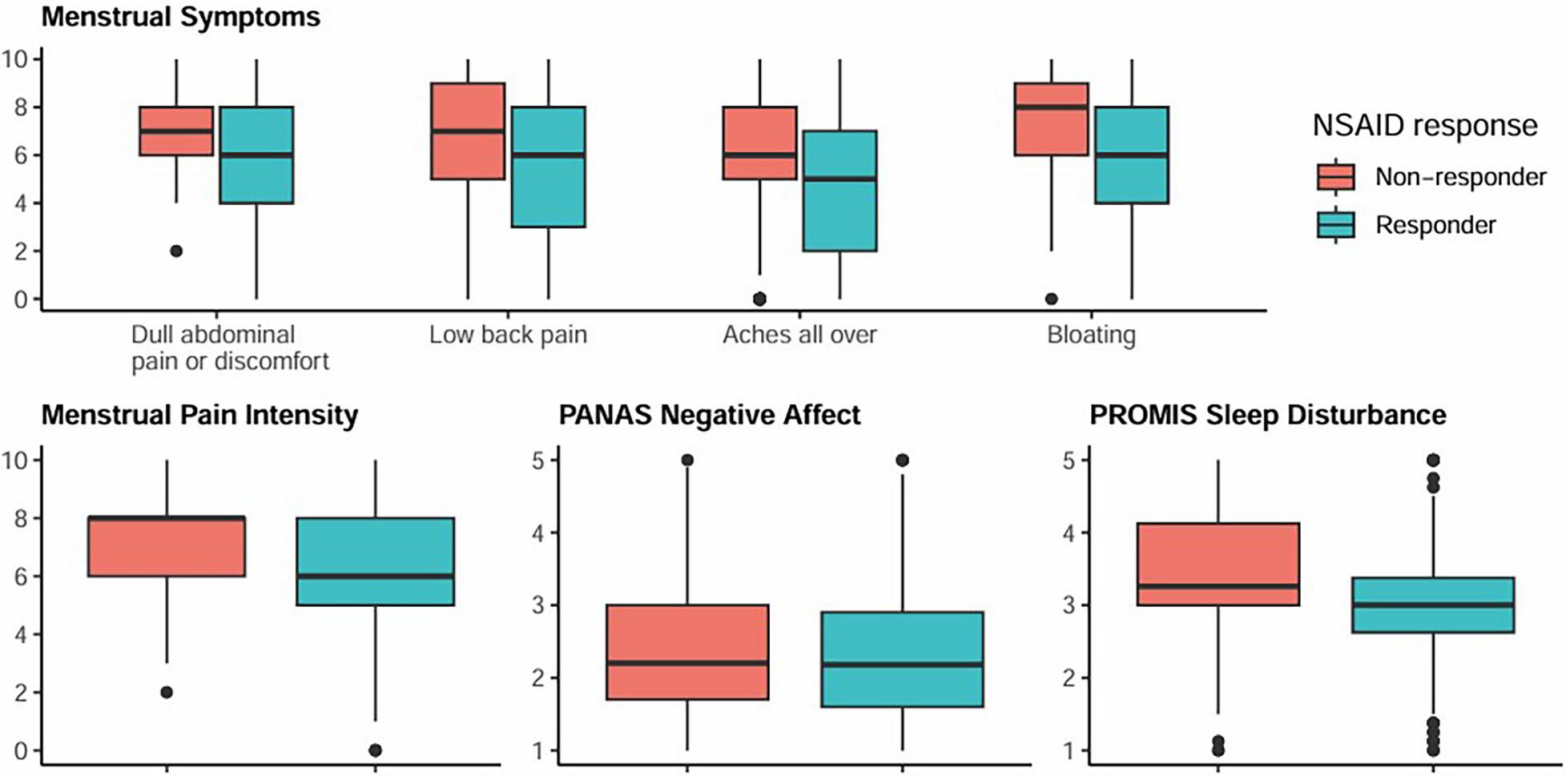
Box plots comparing NSAID responders vs. non-responders on the distributions of seven predictors with selection fractions of 1.00.

Seven predictors were selected in 100% of LASSO models (i.e., selection fraction of 1.00), indicating high stability of results. These most robust factors (with their mean ORs for NSAID response) were typical menstrual pain intensity (OR = 0.75), sleep disturbance (OR = 0.70), negative affect (OR = 1.30), and the following menstrual symptoms: dull abdominal pain or discomfort (OR = 0.82), bloating (OR = 0.76), aches all over (OR = 0.94), and lower back pain (OR = 0.95). In other words, women in our sample who experienced less intense menstrual pain, less severe menstrual symptoms, had lower sleep disturbance, and/or reported more negative mood were more likely to be classified as NSAID responders. Box plots of these seven predictors for NSAID responders and non-responders are shown in Figure 2.

Notably, most variables showed limited or no contribution to the model's predictive accuracy. For example, demographic variables had low effect sizes, and most were not selected in any LASSO model. Menstrual variables that are typically associated with dysmenorrhea (e.g., age of menarche, menstrual bleeding heaviness) were also not included in any model. The Boruta sensitivity analysis similarly classified age at menarche as Rejected (mean importance score = 1.10, below the shadow feature threshold). Clinical variables not selected in any model include specific bodily pain in the past month when not menstruating (e.g., head, neck, knee, low back), other chronic pain conditions (e.g., migraine headaches, fibromyalgia, arthritis), chronic health conditions (e.g., cardiovascular, endocrine, respiratory, or neurological conditions), endometriosis, menstrual sensitivity, depression, anxiety, emotional support, and perceived stress.

### 3.3 Sensitivity analysis shows mixed results

The sensitivity analysis using the alternate coding of the outcome, as described in the Methods section, led to 373 individuals classified as NSAID non-responders, and 139 as NSAID responders. Sensitivity results yielded similar findings for variables with larger effects (i.e., OR) in the main analysis, such as menstrual pain (OR = 0.85), dull abdominal pain or discomfort (OR = 0.91), bloating (OR = 0.76), negative affect (OR = 1.30), and sleep disturbance (OR = 0.73). Otherwise, results were relatively divergent, with notable inclusion of abdominal cramps (OR = 0.67), pain in the upper thighs (OR = 1.11), number of menstrual symptoms endorsed (OR = 0.73), total number of body regions with non-menstrual body pain (OR = 0.83), and the social concerns subscale of the ASI-3 (OR = 1.19). Out of the 25 predictors selected by the main analysis, the sensitivity analysis selected 13. AUC values were identical at 0.64 (standard deviation = 0.01). The full list of the selection fractions and coefficient estimates for each variable, along with the results of the sensitivity analysis, can be found in the Supplementary Materials.

The Boruta algorithm selected 19 predictors, 11 of which were also selected by the main LASSO models. These include menstrual, city, dull abdominal pain or discomfort, low back pain, headache or migraine, aches all over, bloating, reduced appetite, general pain interference, physical function, and sleep disruption. The 8 predictors selected by the Boruta algorithm but not the main LASSO models include period pain interference, abdominal cramps, nausea, more bowel movements, pain in the upper thighs, constipation, anxiety concerning menstrual symptoms, and the number of menstrual symptoms. A full list of the importance values estimated for each predictor can be found in the Supplementary Materials.

## 4 Discussion

In this cross-sectional study of women who use NSAIDs for menstrual pain, we explored a broad array of self-reported factors, identifying those that related to the probability of NSAID response. To attenuate problems associated with traditional approaches while estimating effects for many simultaneous predictors, a machine learning approach to variable selection was used (50). Given the limitations of the data, our findings should be considered hypothesis-generating and suggestive of avenues for further research. Overall, 25 of 116 predictors were selected in at least one of 100 group LASSO models, and seven of those predictors were selected in all 100 models (i.e., selection fraction of 1.00). The largest associations with NSAID response were negative affect (OR = 1.30), number of periods in the past year (OR = 1.11), and the physical concerns factor of anxiety sensitivity (OR = 1.11). The largest associations with NSAID non-response were sleep disturbance (OR = 0.70), menstrual pain severity (OR = 0.75), and bloating (OR = 0.76). In contrast, most psychosocial and clinical variables were not selected by any models and thus were not considered predictive. Gharavi and Mischkowski (20) proposed the hypothesis that psychosocial factors may moderate the effects of pharmacological analgesics, potentially explaining some of the observed response variability. For example, they and others suggest that the observed association of depression and anxiety with higher opioid use (51) points to the possibility of these conditions causing higher dose requirements to achieve pain relief. Interestingly, our results demonstrated that few psychosocial constructs were related to NSAID response, with variables such as companionship, emotional support, depression, and anxiety showing selection fractions of zero.

An unexpected finding was that higher negative affect (recent negative mood) was associated with better odds of NSAID response. It is important to note that though this effect was stable in the main LASSO-based analysis (selection fraction = 1.00, with a standard deviation of 0.06 for the distribution of estimated ORs, and VIF value = 2.15), the effect size was modest (mean OR = 1.30) and was not selected for in the Boruta sensitivity analysis. Thus, this finding should be interpreted with caution. In our sensitivity analysis using an alternate outcome cutpoint (responses 1–3 coded as non-response), negative affect was again selected with a mean OR of 1.30, identical to the primary analysis, suggesting the finding is not an artifact of the specific dichotomization chosen. Interestingly, we did not find a strong association between pain catastrophizing and NSAID response—the PCS magnification subscale showed a very small effect (OR = 1.03) and total PCS scores did not predict NSAID response. This contrasts with prior work showing that catastrophizing is a key determinant of menstrual pain severity and pain interference (52). It may be that while catastrophizing exacerbates the overall pain experience, it does not specifically impede the pharmacological action of NSAIDs on uterine prostaglandin pathways. Women with high negative affect or catastrophizing might report higher baseline menstrual pain, but if that pain is predominantly prostaglandin-driven, NSAIDs could still provide proportionate relief (even if their residual pain remains higher in absolute terms). In other words, psychological distress may raise the pain “floor” but not necessarily the incremental benefit from NSAIDs.

Though some prior research points to evidence of central sensitization being involved in the experience of menstrual pain (e.g. (53, 54),), our findings are mixed regarding whether indices of central sensitization contribute to NSAID response in menstrual pain. Out of 13 possible chronic pain conditions, only low back pain was associated with slightly lower odds of NSAID response, while other variables showed no association with NSAID response, such as menstrual sensitivity, pain catastrophizing, and perceived stress. However, we did find that some factors which likely to contribute to central sensitization were also related to NSAID response, notably with negative affect predicting response to NSAIDs (OR = 1.30), and sleep disturbance predicting non-response to NSAIDs (OR = 0.70). This finding may also reflect limitations in our data—the prevalence of conditions such as fibromyalgia was very low in our sample, and we relied on self-report rather than clinical confirmation. Many studies have found that that young women with dysmenorrhea often have other comorbid pain complaints, but it did not specifically address treatment response (55–57). Our results tentatively suggest that having multiple pain conditions does not automatically render NSAIDs ineffective for menstrual pain, especially if those NSAIDs are targeting a specific prostaglandin-driven mechanism unique to menstruation. Future research should explore additional indices of central sensitization to determine if and how it may be related to response or resistance to NSAIDs.

Our findings also identified higher sleep disturbance as a predictor of NSAID non-response. Generally, the relationship between sleep and chronic pain is complex and likely reciprocal, occurring through multiple mediated pathways (58, 59), with menstrual pain also being associated with sleep impairment (60). An EEG study has shown that severe menstrual pain fragments sleep and reduces sleep efficiency, with promising effects for NSAIDs in improving both menstrual pain and sleep quality (61). Though our cross-sectional data and modeling approach cannot determine causality or its direction, this finding suggests that assessing sleep may be important in dysmenorrhea management and that adjunctive strategies aimed at improving sleep (or using bedtime analgesic dosing) could also be beneficial for NSAID non-responders.

The Boruta sensitivity analysis did not show complete consistency with the main LASSO-based analyses. There are a few reasons why this may be the case. Firstly, in the presence of highly collinear predictors, LASSO regularization will tend to select one and discard the others whereas the Boruta (based on random forests) will select all predictors that contribute to predictive accuracy. This suggests that variables selected by the Boruta algorithm but not the LASSO models may carry redundant information in terms of predictive modeling, but may be clinically interesting regardless. Secondly, LASSO regularization selects predictors that have direct and linear associations with the outcome, while the Boruta algorithm automatically includes predictors with complex non-linear and interaction effects. Thus, the variables exclusively selected by the LASSO may demonstrate small and unstable linear effects which the Boruta algorithm deemed unimportant, and variables selected exclusively by the Boruta algorithm may carry strictly non-linear and interaction effects. The decision on which variable selection method to use may be influenced by factors such as the desire for a parsimonious vs. all relevant set of predictors, the detection of complex non-linear relationships, and access to interpretable coefficient estimates. The absence of a formally held-out independent test set is a shared limitation of both the LASSO and Boruta approaches; cross-validated AUC estimates should be interpreted with awareness that some degree of optimism may remain.

Key strengths of our study include the large sample size, use of advanced machine learning methods, and comprehensive set of variables examined, ranging from clinical to validated psychosocial domains. A few limitations to the study must be noted. First, this study uses an exploratory approach to identify which factors may be relevant to NSAID response in the context of little existing theory to guide *a priori* variable selection. Thus, findings presented in this paper should be considered tentative, requiring further hypothesis testing. Second, associations revealed from observational cross-sectional data do not necessarily constitute evidence of direct causal relationships, as many different causal structures can manifest in identical observed covariances. Third, our data was self-report, including the outcome measure (perceived NSAID response), which is subjective and may be influenced by individual expectations or placebo/nocebo effects. We did not verify actual medication use, nor did we collect information on specific NSAID types and dosing regimens—different NSAIDs or suboptimal dosing could have varying success rates. Different NSAIDs have distinct pharmacokinetic profiles and varying potency at inhibiting prostaglandin synthesis, and suboptimal dosing or delayed initiation relative to menstrual onset could substantially reduce perceived efficacy independent of true pharmacological resistance. As a result, some participants classified as non-responders may represent inadequate drug exposure rather than failure of the prostaglandin mechanism. Future prospective studies should capture NSAID name, dose, timing relative to pain onset, and adherence to enable more precise characterization of pharmacological non-response. Further, our sample may under-represent women with secondary dysmenorrhea and very severe pain who have already escalated to other treatments or who do not use NSAIDs at all. In fact, by requiring that participants use NSAIDs, we inherently excluded those who found NSAIDs completely ineffective in the past and abandoned them. This selection bias means our “non-responder” group may not include the absolute worst cases of NSAID failure, and thus may underestimate the true prevalence of NSAID resistance in the general menstruating population It may also shift predictor identification toward variables distinguishing partial from complete responders, rather than responders from treatment-refractory cases. Findings should therefore be interpreted with this restriction in mind. For example, the low prevalence of endometriosis in our sample and the known underdiagnosis of this condition suggests limited ability of our study to detect associations of NSAID non-response in secondary dysmenorrhea. Self-reported physician diagnosis substantially underestimates true prevalence of endometriosis, and the unknown proportion of undiagnosed cases limits detection of associations between secondary dysmenorrhea and NSAID non-response. The selection bias described above further compounds this: women with endometriosis who had already escalated to alternative treatments or abandoned NSAIDs entirely would have been excluded, making this subgroup particularly under-represented in the current sample. Lastly, our sample only consists of adults, and thus would not generalize to menstrual pain conditions associated with adolescence and menarche. Additionally, calibration performance beyond discrimination (e.g., Brier score, calibration slope) was not formally assessed in this exploratory analysis; this would be an important consideration before any future effort to develop a clinically deployable prediction model from these findings. Our results should therefore be considered hypothesis-generating and relevant primarily to general populations rather than specialized pain clinic samples.

### 4.1 Conclusion

Though NSAIDs remain a first-line therapy for dysmenorrhea due to demonstrated efficacy, 20% of patients experience incomplete pain relief from NSAIDs alone (12). In this exploratory study, we found that women with menstrual pain who respond poorly to NSAIDs are characterized by higher menstrual pain intensity, more severe menstrual symptoms, and greater sleep disturbances, suggesting a phenotype of overall amplified pain experience. Our findings reinforce that addressing co-factors (poor sleep, etc.) should be part of dysmenorrhea management. Ongoing research is needed to unravel the biological mechanisms of NSAID resistance and to develop targeted interventions for those women who continue to suffer menstrual pain despite standard therapies. By identifying who is at risk for NSAID non-response and why, we can move toward more individualized and effective management of dysmenorrhea, ultimately reducing the significant impact this condition has on women's lives.

## Data Availability

The datasets presented in this article are not readily available because the de-identified data that support the findings of this study are available on request from the corresponding author. The current approved institutional mechanism for data sharing is by individual data use agreements executed between the interested parties. Requests to access the datasets should be directed to Laura Payne L Payne@mclean.harvard.edu.

## References

[1] Ju H Jones M Mishra G. The prevalence and risk factors of dysmenorrhea. Epidemiol Rev. (2014) 36(1):104–13. 10.1093/epirev/mxt009 24284871

[2] Sims H Singh B. The impact of menorrhagia/dysmenorrhea on work performance. Women’s Reprod Health. (2024) 11(1):166–81. 10.1080/23293691.2023.2209077

[3] Armour M Parry K Manohar N Holmes K Ferfolja T Curry C. The prevalence and academic impact of dysmenorrhea in 21,573 young women: a systematic review and meta-analysis. J Womens Health. (2019) 28(8):1161–71. 10.1089/jwh.2018.7615 31170024

[4] Hellman KM Kuhn CS Tu FF Dillane KE Shlobin NA Senapati S. Cine MRI during spontaneous cramps in women with menstrual pain. Am J Obstet Gynecol. (2018) 218(5):506–e1. 10.1016/j.ajog.2018.01.035 PMC5916049 29409786

[5] Kantarovich D Dillane KE Garrison EF Oladosu FA Schroer MS Roth GE. Development and validation of a real-time method characterizing spontaneous pain in women with dysmenorrhea. J Obstet Gynaecol Res. (2021) 47(4):1472–80. 10.1111/jog.14663 33590541 PMC8317258

[6] Oladosu FA Tu FF Hellman KM. Nonsteroidal antiinflammatory drug resistance in dysmenorrhea: epidemiology, causes, and treatment. Am J Obstet Gynecol. (2018) 218(4):390–400. 10.1016/j.ajog.2017.08.108 28888592 PMC5839921

[7] Kyathanahalli CN Tu FF Hellman KM. Inflammatory mechanisms of dysmenorrhea: novel insights from menstrual effluent in an adolescent cohort. BJOG. (2025) 10:132:1626–34. 10.1111/1471-0528.18275 40631483 PMC12258968

[8] Payne LA Rapkin AJ Seidman LC Zeltzer LK Tsao JC. Experimental and procedural pain responses in primary dysmenorrhea: a systematic review. J Pain Res. (2017):2233–46. 10.2147/JPR.S143512 29066929 PMC5604431

[9] Marjoribanks J Ayeleke RO Farquhar C Proctor M. Nonsteroidal anti-inflammatory drugs for dysmenorrhoea. Cochrane Database Syst Rev. (2015) 2015(7):CD001751. 10.1002/14651858.CD001751.pub3 26224322 PMC6953236

[10] Hellman KM Roth GE Dillane KE Garrison EF Oladosu FA Clauw DJ. Dysmenorrhea subtypes exhibit differential quantitative sensory assessment profiles. Pain. (2020) 161(6):1227–36. 10.1097/j.pain.0000000000001826 32168005 PMC7230023

[11] Milsom I Minic M Dawood MY Akin MD Spann J Niland NF. Comparison of the efficacy and safety of nonprescription doses of naproxen and naproxen sodium with ibuprofen, Acetaminophen, and placebo in the treatment of primary dysmenorrhea: a pooled analysis of five studies. Clin Ther. (2002) 24(9):1384–400. 10.1016/S0149-2918(02)80043-1 12380631

[12] Owen PR. Prostaglandin synthetase inhibitors in the treatment of primary dysmenorrhea: outcome trials reviewed. Am J Obstet Gynecol. (1984) 148(1):96–103. 10.1016/S0002-9378(84)80039-3 6419611

[13] Oladosu FA Tu FF Garrison EF Dillane KE Roth GE Hellman KM. Low serum naproxen concentrations are associated with minimal pain relief: a preliminary study in women with dysmenorrhea. Pain Medicine. (2020) 21(11):3102–8. 10.1093/pm/pnaa133 32488234 PMC7784249

[14] Brown J Crawford TJ Allen C Hopewell S Prentice A. Nonsteroidal anti-inflammatory drugs for pain in women with endometriosis. Cochrane Database Syst Rev. (2017) 1(1):CD004753. 10.1002/14651858.CD004753.pub4 PMC6464974 28114727

[15] Woolf CJ. Central sensitization: implications for the diagnosis and treatment of pain. pain. (2011) 152(3):S2–15. 10.1016/j.pain.2010.09.030 20961685 PMC3268359

[16] Payne LA Seidman LC Sim MS Rapkin AJ Naliboff BD Zeltzer LK. Experimental evaluation of central pain processes in young women with primary dysmenorrhea. Pain. (2019) 160(6):1421–30. 10.1097/j.pain.0000000000001516 30720583 PMC6527468

[17] Nijs J Meeus M Van Oosterwijck J Roussel N De Kooning M Ickmans K. Treatment of central sensitization in patients with ‘unexplained’chronic pain: what options do we have? Expert Opin Pharmacother. (2011) 12(7):1087–98. 10.1517/14656566.2011.547475 21254866

[18] Gagnon MM Moussaoui D Gordon JL Alberts NM Grover SR. Dysmenorrhea across the lifespan: a biopsychosocial perspective to understanding the dysmenorrhea trajectory and association with comorbid pain experiences. Pain. (2022) 163(11):2069–75. 10.1097/j.pain.0000000000002649 35420567

[19] Chen CX Rogers SK Li R Hinrichs RJ Fortenberry JD Carpenter JS. Social determinants of health and dysmenorrhea: a systematic review. J Pain. (2024) 25(9):104574. 10.1016/j.jpain.2024.104574 38788887 PMC11347097

[20] Gharavi E Mischkowski D. On the role of psychological and social factors in pharmacological analgesia: a psychosocial moderation hypothesis. Psychol Rev. (2026) 133(1):197–220. 10.1037/rev0000536 39946610

[21] Trépanier LC Lamoureux É Bjornson SE Mackie C Alberts NM Gagnon MM. Smartphone apps for menstrual pain and symptom management: a scoping review. Internet Interv. (2023) 31:100605. 10.1016/j.invent.2023.100605 36761398 PMC9905939

[22] Payne LA Seidman LC Ren B Greenfield SF. COVID-related distress is associated with increased menstrual pain and symptoms in adult women. Int J Environ Res Public Health. (2022) 20(1):774. 10.3390/ijerph20010774 36613098 PMC9819102

[23] Handy AB Seidman LC Payne LA. Development and initial validation of the menstrual sensitivity index. Pain Medicine. (2024) 25(1):78–85. 10.1093/pm/pnad124 37688582 PMC10765159

[24] Dworkin RH Turk DC Farrar JT Haythornthwaite JA Jensen MP Katz NP. core outcome measures for chronic pain clinical trials: iMMPACT recommendations. pain. (2005) 113(1–2):9–19. 10.1016/j.pain.2004.09.012 15621359

[25] Harris PA Taylor R Thielke R Payne J Gonzalez N Conde JG. Research electronic data capture (REDCap)—a metadata-driven methodology and workflow process for providing translational research informatics support. J Biomed Inform. (2009) 42(2):377–81. 10.1016/j.jbi.2008.08.010 18929686 PMC2700030

[26] Harris PA Taylor R Minor BL Elliott V Fernandez M O’Neal L. The REDCap consortium: building an international community of software platform partners. J Biomed Inform. (2019) 95:103208. 10.1016/j.jbi.2019.103208 31078660 PMC7254481

[27] Chen CX Ofner S Bakoyannis G Kwekkeboom KL Carpenter JS. Symptoms-based phenotypes among women with dysmenorrhea: a latent class analysis. West J Nurs Res. (2018) 40(10):1452–68. 10.1177/0193945917731778 28914180 PMC5832523

[28] Sullivan MJ Bishop SR Pivik J. The pain catastrophizing scale: development and validation. Psychol Assess. (1995) 7(4):524. 10.1037/1040-3590.7.4.524

[29] Watson D Clark LA Tellegen A. Development and validation of brief measures of positive and negative affect: the PANAS scales. J Pers Soc Psychol. (1988) 54(6):1063. 10.1037/0022-3514.54.6.1063 3397865

[30] Cohen S. Perceived stress in a probability sample of the United States (1988).

[31] Taylor S Zvolensky MJ Cox BJ Deacon B Heimberg RG Ledley DR. Robust dimensions of anxiety sensitivity: development and initial validation of the anxiety sensitivity Index-3. Psychol Assess. (2007) 19(2):176. 10.1037/1040-3590.19.2.176 17563199

[32] Cella D Riley W Stone A Rothrock N Reeve B Yount S. The patient-reported outcomes measurement information system (PROMIS) developed and tested its first wave of adult self-reported health outcome item banks: 2005–2008. J Clin Epidemiol. (2010) 63(11):1179–94. 10.1016/j.jclinepi.2010.04.011 20685078 PMC2965562

[33] Pilkonis PA Choi SW Reise SP Stover AM Riley WT Cella D. Item banks for measuring emotional distress from the patient-reported outcomes measurement information system (PROMIS®): depression, anxiety, and anger. Assessment. (2011) 18(3):263–83. 10.1177/1073191111411667 21697139 PMC3153635

[34] Pilkonis PA Yu L Dodds NE Johnston KL Maihoefer CC Lawrence SM. Validation of the depression item bank from the patient-reported outcomes measurement information system (PROMIS®) in a three-month observational study. J Psychiatr Res. (2014) 56:112–9. 10.1016/j.jpsychires.2014.05.010 24931848 PMC4096965

[35] Amtmann D Cook KF Jensen MP Chen WH Choi S Revicki D. Development of a PROMIS item bank to measure pain interference. Pain. (2010) 150(1):173–82. 10.1016/j.pain.2010.04.025 20554116 PMC2916053

[36] Rose M Bjorner JB Gandek B Bruce B Fries JF Ware Jr JE. The PROMIS physical function item bank was calibrated to a standardized metric and shown to improve measurement efficiency. J Clin Epidemiol. (2014) 67(5):516–26. 10.1016/j.jclinepi.2013.10.024 24698295 PMC4465404

[37] Hahn EA DeWalt DA Bode RK Garcia SF DeVellis RF Correia H. New English and Spanish social health measures will facilitate evaluating health determinants. Health Psychol. (2014) 33(5):490. 10.1037/hea0000055 24447188 PMC4159098

[38] Lai JS Cella D Choi S Junghaenel DU Christodoulou C Gershon R. How item banks and their application can influence measurement practice in rehabilitation medicine: a PROMIS fatigue item bank example. Arch Phys Med Rehabil. (2011) 92(10):S20–7. 10.1016/j.apmr.2010.08.033 21958919 PMC3696589

[39] Buysse DJ Yu L Moul DE Germain A Stover A Dodds NE. Development and validation of patient-reported outcome measures for sleep disturbance and sleep-related impairments. Sleep. (2010) 33(6):781–92. 10.1093/sleep/33.6.781 20550019 PMC2880437

[40] Yu L Buysse DJ Germain A Moul DE Stover A Dodds NE. Development of short forms from the PROMIS^TM^ sleep disturbance and sleep-related impairment item banks. Behav Sleep Med. (2012) 10(1):6–24. 10.1080/15402002.2012.636266 PMC3261577 22250775

[41] Babyak MA. What you see may not be what you get: a brief, nontechnical introduction to overfitting in regression-type models. Biopsychosoc Sci Med. (2004) 66(3):411–21. 10.1097/01.psy.0000127692.23278.a9 15184705

[42] Tibshirani R. Regression shrinkage and selection via the lasso. J R Stat Soc Ser B Stat Methodol. (1996) 58(1):267–88. 10.1111/j.2517-6161.1996.tb02080.x

[43] McNeish DM. Using lasso for predictor selection and to assuage overfitting: a method long overlooked in behavioral sciences. Multivariate Behav Res. (2015) 50(5):471–84. 10.1080/00273171.2015.1036965 26610247

[44] Yang Y Zou H. A fast unified algorithm for solving group-lasso penalize learning problems. Stat Comput. (2015) 25(6):1129–41. 10.1007/s11222-014-9498-5

[45] Yang Y Zou H Bhatnagar S. gglasso: Group Lasso Penalized Learning Using a Unified BMD Algorithm [Internet] (2024). Available online at: https://CRAN.R-project.org/package=gglasso (Accessed August 05, 2025).

[46] Meier L Van De Geer S Bühlmann P. The group lasso for logistic regression. J R Stat Soc Ser B Stat Methodol. (2008) 70(1):53–71. 10.1111/j.1467-9868.2007.00627.x

[47] Han SYS Cooper JD Ozcan S Rustogi N Penninx BW Bahn S. Integrating proteomic, sociodemographic and clinical data to predict future depression diagnosis in subthreshold symptomatic individuals. Transl Psychiatry. (2019) 9(1):277. 10.1038/s41398-019-0623-2 31699963 PMC6838310

[48] Stekhoven DJ Bühlmann P. Missforest—non-parametric missing value imputation for mixed-type data. Bioinformatics. (2012) 28(1):112–8. 10.1093/bioinformatics/btr597 22039212

[49] Kursa MB, Rudnicki WR. Feature selection with the boruta Package. *J Stat Softw*. (2010) 36(11):1–13. 10.18637/jss.v036.i11

[50] Greenwood CJ Youssef GJ Letcher P Macdonald JA Hagg LJ Sanson A. A comparison of penalised regression methods for informing the selection of predictive markers. PLoS One. (2020) 15(11):e0242730. 10.1371/journal.pone.0242730 33216811 PMC7678959

[51] Rogers AH Orr MF Shepherd JM Bakhshaie J Ditre JW Buckner JD. Anxiety, depression, and opioid misuse among adults with chronic pain: the role of emotion dysregulation. J Behav Med. (2021) 44(1):66–73. 10.1007/s10865-020-00169-8 32594288 PMC11862901

[52] Suso-Ribera C García-Palacios A Botella C Ribera-Canudas MV. Pain catastrophizing and its relationship with health outcomes: does pain intensity matter? Pain Res Manag. (2017) 2017(1):9762864. 10.1155/2017/9762864 28348506 PMC5350380

[53] Hoyos-Calderon YT Martínez-Merinero P Nunez-Nagy S Pecos-Martín D Calvo-Lobo C Romero-Morales C. Myofascial trigger points and central sensitization signs, but no anxiety, are shown in women with dysmenorrhea: a case-control study. Biology (Basel). (2022) 11(11):1550. 10.3390/biology11111550 36358253 PMC9688021

[54] Vincent K Warnaby C Stagg CJ Moore J Kennedy S Tracey I. Dysmenorrhoea is associated with central changes in otherwise healthy women. Pain. (2011) 152(9):1966–75. 10.1016/j.pain.2011.03.029 21524851

[55] Westling AM Tu FF Griffith JW Hellman KM. The association of dysmenorrhea with noncyclic pelvic pain accounting for psychological factors. Am J Obstet Gynecol. (2013) 209(5):422–e1. 10.1016/j.ajog.2013.08.020 PMC4191839 23973396

[56] Li R Li B Kreher DA Benjamin AR Gubbels A Smith SM. Association between dysmenorrhea and chronic pain: a systematic review and meta-analysis of population-based studies. Am J Obstet Gynecol. (2020) 223(3):350–71. 10.1016/j.ajog.2020.03.002 32151612

[57] Zondervan KT Yudkin PL Vessey MP Jenkinson CP Dawes MG Barlow DH. Chronic pelvic pain in the community—symptoms, investigations, and diagnoses. Am J Obstet Gynecol. (2001) 184(6):1149–55. 10.1067/mob.2001.112904 11349181

[58] Finan PH Goodin BR Smith MT. The association of sleep and pain: an update and a path forward. J Pain. (2013) 14(12):1539–52. 10.1016/j.jpain.2013.08.007 24290442 PMC4046588

[59] Whibley D AlKandari N Kristensen K Barnish M Rzewuska M Druce KL. Sleep and pain: a systematic review of studies of mediation. Clin J Pain. (2019) 35(6):544–58. 10.1097/AJP.0000000000000697 30829737 PMC6504189

[60] Shaver JL Iacovides S. Sleep in women with chronic pain and autoimmune conditions: a narrative review. Sleep Med Clin. (2018) 13(3):375–94. 10.1016/j.jsmc.2018.04.008 30098754

[61] Iacovides S Avidon I Bentley A Baker FC. Diclofenac potassium restores objective and subjective measures of sleep quality in women with primary dysmenorrhea. Sleep. (2009) 32(8):1019–26. 10.1093/sleep/32.8.1019 19725253 PMC2717192

